# Robust deep MRI contrast synthesis using a prior-based and task-oriented 3D network

**DOI:** 10.1162/IMAG.a.116

**Published:** 2025-08-26

**Authors:** Sergio Morell-Ortega, Marina Ruiz-Perez, Marien Gadea, Roberto Vivo-Hernando, Gregorio Rubio, Fernando Aparici, Mariam de la Iglesia-Vaya, Thomas Tourdias, Boris Mansencal, Pierrick Coupé, José V. Manjón

**Affiliations:** Instituto de Aplicaciones de las Tecnologías de la Información y de las Comunicaciones Avanzadas (ITACA), Universitat Politècnica de València, Valencia, Spain; Department of Psychobiology, Faculty of Psychology, Universitat de València, Valencia, Spain; Instituto de Automática e Informática Industrial, Universitat Politècnica de València, Valencia, Spain; Departamento de matemática aplicada, Universitat Politècnica de València, Valencia, Spain; Área de Imagen Médica, Hospital Universitario y Politécnico La Fe, Valencia, Spain; Unidad Mixta de Imagen Biomédica FISABIO-CIPF, Fundación para el Fomento de la Investigación Sanitario y Biomédica de la Comunidad Valenciana—Valencia, Spain; Univ. Bordeaux, INSERM, Neurocentre Magendie, U1215, Bordeaux, France; CNRS, Univ. Bordeaux, Bordeaux INP, LABRI, UMR5800, in2brain, Talence, France

**Keywords:** MRI, contrast synthesis, deep learning, semi-supervised learning

## Abstract

Magnetic resonance imaging (MRI) is one of the most widely used tools for clinical diagnosis. Depending on the acquisition parameters, different image contrasts can be obtained, providing complementary information about the patient’s anatomy and potential pathological findings. However, multiplying such acquisitions requires more time, additional resources, and increases patient discomfort. Consequently, not all image modalities are typically acquired. One solution to obtain the missing modalities is to use contrast synthesis methods. Most existing synthesis methods work with 2D slices due to memory limitations, which produces inconsistencies and artifacts when reconstructing the 3D volume. In this work, we present a 3D deep learning-based approach for synthesizing T2-weighted MR volumes from T1-weighted ones. To preserve anatomical details and enhance image quality, we propose a segmentation-oriented loss function combined with a frequency space information loss. To make the proposed method more robust and applicable to a wider range of image scenarios, we also incorporate a priori information in the form of a multi-atlas. Additionally, we employ a semi-supervised learning framework that improves the model’s generalizability across diverse datasets, potentially improving its performance in clinical settings with varying patient demographics and imaging protocols. By integrating prior anatomical knowledge with frequency domain and segmentation loss functions, our approach outperforms state-of-the-art methods, particularly in segmentation tasks. The method demonstrates significant improvements, especially in challenging cases, compared with state-of-the-art approaches.

## 1 Introduction

Magnetic resonance imaging (MRI) can noninvasively acquire images with different contrasts of the same anatomical region, with each contrast providing complementary information. For instance, T1-weighted (T1) images clearly differentiate brain gray matter from white matter, while T2-weighted (T2) images distinguish cortical gray matter from cerebrospinal fluid, and FLAIR imaging assists in lesion detection.

The literature confirms that combining T1 and T2 MRI improves downstream tasks. For the segmentation of brain anatomy or lesions, multimodal pipelines (T1 + T2/FLAIR) greatly reduce misclassification errors (Lindig et al., 2018; Morell-Ortega et al., 2025; Ruiz-Perez et al., 2024; Viviani et al., 2017). In disease classification and grading, adding T2 data significantly improves accuracy and AUC (Zeng et al., 2024). Besides, in image reconstruction, the use of multimodal information helps to recover high-quality images from limited data (Xiang et al., 2019).

However, acquiring different image contrasts requires modifying the MR acquisition parameters and obtaining additional images. This extended acquisition process is costly, time consuming, resource intensive, and increases patient discomfort. Consequently, in recent years there has been growing interest in automatic image synthesis as an efficient way to enhance image analysis (e.g., brain tissue segmentation; Manjón et al., 2022) at a lower acquisition cost.

For example, Iglesias et al. (2013) were among the first to propose image synthesis to enable the use of Freesurfer software by synthesizing T1 images from original T2 images, since the software was originally designed to work exclusively with T1 images.

Earlier methods for image synthesis included non-linear registration-based (Burgos et al., 2014; Miller et al., 1993) and intensity transformation-based methods (Iglesias et al., 2013; Jog et al., 2017; Roy et al., 2013). More recently, Deep learning techniques have emerged as a method capable of learning non-linear mappings for image synthesis. A recent review by Dayarathna et al. (2024), provides a comprehensive analysis of widely used methods, offering valuable insights into the current state of the field.

The most commonly used networks for image-to-image translation are based on GANs (Generative Adversarial Networks), as for example, the work done in Dar et al. (2019), where they also leverage correlated 2D information across neighboring cross sections within a volume. These methods used mainly 2D approaches due to the memory overhead of their generator–discriminator approach (Dar et al., 2019; Liu et al., 2021), but there are also 3D patch-based GAN methods that deal with the high memory consumption using small volume patches (Uzunova et al., 2020; Zhao et al., 2021).

Various models have been employed for the generator architecture. For example, the well-known U-Net and variants have been used in several methods (Manjón et al., 2022; Osman & Tamam, 2022). More advanced architectures, including recent methods based on vision transformers, have also been proposed (Dalmaz et al., 2022; Y. Li et al., 2022; J. Liu et al., 2022; K. Pan et al., 2022; Yan et al., 2022).

The most recent methods for image synthesis using deep learning are based on diffusion models due to their high ability to sample the target distribution. Meng et al. (2022) proposed the Unified Multi-Modal Conditional Score-based Generative Model (UMM-CSGM), using conditional diffusion and reverse generation in the complete modality space. Therefore, they can learn a comprehensive set of cross-modal conditional distributions. Due to promising results in diffusion, Yoon et al. (2023) proposed a sequence-aware diffusion model (SADM) to generate longitudinal medical images. This design facilitates learning longitudinal dependencies even in the presence of missing data during training and supports the autoregressive generation of image sequences during inference. Pan et al. (2023) developed a novel Cycle-guided Denoising Diffusion Probability Model (CG-DDPM) for cross-modality MRI synthesis. It utilizes two interconnected DDPMs that condition one another to generate synthetic images from two distinct MRI pulse sequences. During the reverse processes, the two DDPMs exchange random latent noise, which serves to regularize both models and produce consistent images across the two modalities. This approach enhances the accuracy of image-to-image translation. Arslan et al. (2024) proposed a novel self-consistent recursive diffusion bridge (SelfRDB) that employs an innovative noise scheduling strategy in its forward process, where the variance progressively increases until reaching the end point, representing a noise-enhanced source image.

Other studies have explored untrained generative priors (e.g., deep image priors; Ulyanov et al., 2018) and learned diffusion priors for medical images, which can provide structured regularization in reconstruction and synthesis tasks. Besides, hybrid architectures that embed generative modules or physical models into neural networks have also been proposed. For example, Atli et al. (2024) introduced I2I-Mamba, a hybrid state-space/CNN model for multi-modal MRI synthesis.

As noted, most image synthesis approaches are 2D-based or patch-based methods. However, it is well known that 3D methods are generally more consistent, accurate, and efficient (Crespi et al., 2022; Shivdeo et al., 2021). To reduce memory consumption, approaches such as 2D to 3D mapping (Zhu et al., 2023) or 3D spatial-to-depth encoding (Manjón et al., 2022) have been used. Manjón et al. (2022) proposed a modified version of U-Net designed to handle entire 3D volumes. This method works by splitting the input volume into eight smaller sub-volumes using a decimation technique in a mosaic pattern. This approach enables the use of a greater number of filters, enhancing the model’s capability to process the data effectively. Zhang et al. (2022) introduced a novel 3D patch-based MRI synthesis framework and a pyramid transformer network (PTNet3D) that relies on attention mechanisms implemented through transformer layers.

In summary, many of the proposed methods rely on GANs or diffusion models, which can generate highly realistic results. However, GANs have the risk of hallucinating structural details, potentially introducing artifacts in the generated images (Teneggi et al., 2023). However, diffusion models, despite delivering excellent results, are often time consuming due to their iterative nature (Teneggi et al., 2023). Moreover, both approaches typically use 2D techniques because their complexity and memory demands make infeasible to work in 3D, which can lead to inconsistencies across 3D slices, compromising the continuity and accuracy of the generated images.

In this work, we propose a task-oriented approach for synthesizing 3D T2 MR images from T1 ones. Our method uses a lightweight 3D architecture specifically designed for synthesis tasks, enhanced by prior information in the form of a multi-atlas derived from real T2 images. Additionally, we explored both global and local loss terms—such as spatial, frequency-based, and segmentation-task losses—to ensure consistency, precision, and effectiveness in the regression task. Furthermore, we implemented a semi-supervised approach to increase the variability of the training set, thereby enhancing the robustness of the final model.

## 2 Methods

### 2.1 Datasets

This study utilizes six distinct datasets. Because images captured with different scanners or acquisition parameters naturally exhibit varying characteristics, incorporating multiple databases helps enhance the model’s robustness to these variations (four of the datasets include paired T1 and T2 MR brain images). These datasets are:

- **HCP dataset**: This dataset consists of 1,104 T1/T2 MR images from the Human Connectome Project (HCP) (Van Essen et al., 2012). This dataset consists of MR images taken on a 3T machine from healthy subjects aged between 22 and 35 years. For T1-weighted imaging, the acquisition was 3D MPRAGE (TR = 2,400 ms, TE = 2.14 ms, TI = 1,000 ms, Flip Angle: 8°, FOV = 224 mm × 224 mm, Voxel Size: 0.7 mm isotropic, BW = 210 Hz/Px, iPAT = Factor of 2, Acquisition Time: Approximately 7 minutes and 40 seconds). For T2-weighted imaging, the sequence is 3D T2-SPACE (TR = 3,200 ms, TE = 565 ms, Flip Angle: Variable, FOV = 224 mm × 224 mm, Voxel Size: 0.7 mm isotropic, BW = 744 Hz/Px, iPAT: Factor of 2, Acquisition Time: Approximately 8 minutes and 24 seconds).
- **IXI dataset**: This dataset consists of 580 T1/T2 MR images from the IXI Project (https://brain-development.org/ixi-dataset/) acquired in 1.5T and 3T machines. The 3T T1 images were acquired on a Philips Intera 3T scanner (TR = 9.6 ms, TE = 4.6 ms, flip angle = 8°, volume size = 256 × 256 × 150 voxels, voxel dimensions = 0.94 × 0.94 × 1.2 mm^3^). The 1.5T T1 images were acquired on a Philips Gyroscan 1.5T scanner (TR = 9.8 ms, TE = 4.6 ms, flip angle = 8°, slice thickness = 1.2 mm, volume size = 256 × 256 × 150, voxel dimensions = 0.94 × 0.94 × 1.2 mm^3^). The parameters of the 3T T2 images were (TR = 5,725 ms, TE = 100 ms, flip angle = 90º, volume size = 192 × 187 × 187 voxels, voxel dimensions = 0.94 × 0.94 × 1.2 mm^3^) and those for the 1.5T images were (TR = 8,178 ms, TE = 100 ms, flip angle = 90º, volume size = 192 × 187 × 187 voxels, voxel dimensions = 0.94 × 0.94 × 1.2 mm^3^). Images from subjects presenting excessive artifacts or inconsistences with their corresponding paired image were excluded using a correlation analysis resulting in the final number of subjects used of 420. The age range of the subjects of this dataset is between 24 and 75 years.
- **OpenNeuro dataset**: This dataset comes from OpenNeuro platform (https://OpenNeuro.org). It contains 269 T1 and T2 MR images from various projects. Details can be found on their specific papers (Hanke et al., 2018; Rogers et al., 2022; Pinho et al., 2018; Racey et al., 2023; Wolna & Wodniecka, 2023). The age range of the subjects of this dataset is between 19 and 81 years.
- **Bordeaux dataset**: This dataset consists of 44 T1/T2 MR images from a private dataset acquired in a 3T scanner (Vantage Galan 3T/ZGO; Canon Medical Systems) in Bordeaux Hospital as a part of the DeepMultiBrain research project. In this dataset, T1 and T2 images had a matrix size of 256 x 376 x 368 voxels and a voxel size of 0.6 x 0.6 x 0.6 mm^3^. The age range of the subjects of this dataset is between 20 and 64 years.
- **OASIS1 dataset**: This dataset contains 416 T1 MR images from Open Access Series of Imaging Studies (OASIS) (https://sites.wustl.edu/oasisbrains/). We randomly select a set of 100 cases. The MPRAGE images (TR = 9.7 ms, TE = 4 ms, TI = 20 ms, flip angle = 10º, slice thickness = 1.25 mm, matrix size = 256 x 256, voxel dimensions = 1 x 1 x 1.25 mm^3^ resliced to 1 mm^3^, averages = 1) were acquired on a 1.5T Vision scanner (Siemens, Erlangen, Germany). The age range of the subjects of this dataset is between 18 and 96 years.
- **Lifespan dataset**: This last dataset is made of a set of quality curated 3,067 standard resolution T1 images from various public databases (age from 3 to 90 years) previously used in a project to construct a lifespan model of the human brain. Details can be found in Coupé et al. (2017). These databases are the following:
  1. NDAR (N = 403): The Database for Autism Research (NDAR) is a national database funded by NIH (https://ndar.nih.gov). This database included 13 different MRI cohorts acquired on 1.5T and 3T scanners. In our study, we used 206 images of control subjects from the NIHPD (https://www.bic.mni.mcgill.ca/nihpd_info/info2/data_access.html) dataset and 197 images of control subjects from the Lab Study 19 of National Database for Autism Research. For the NIHPD, T1-weighted images were acquired at six different sites with 1.5T systems by General Electric and Siemens Medical Systems. The MR images are 3D T1-weighted spoiled gradient recalled (SPGR) echo sequence (the parameters are TR = 22–25 ms, TE = 10–11 ms, flip angle = 30º, FoV= 256 mm IS x 256 mm AP, matrix size = 256 x 256: 1 x 1 x 1 mm^3^ voxels, 160–180 slices of sagittal orientation). The participants chosen from the Lab Study 19 of National Database for Autism Research (NDAR) were scanned using a 3T Siemens Tim Trio scanner at each site. The MR images are 3D MPRAGE sequence (voxel dimensions: 1.0 x 1.0 x 1.0 mm^3^; image dimensions: 160 x 224 x 256, TE = 3.16 ms, TR = 2,400 ms).
  2. AIBL (N = 343): The Australian Imaging, Biomarkers and Lifestyle (AIBL) dataset. The imaging protocol was defined to follow ADNI’s guideline on the 3T scanner (https://adni.loni.usc.edu/data-samples/adni-data/neuroimaging/mri/mri-scanner-protocols/) and a custom MPRAGE sequence was used on the 1.5T scanner.
  3. ADNI (N = 1,772): The images from the Alzheimer’s Disease Neuroimaging Initiative (ADNI) dataset consisted of subjects from the 1.5T and 3T MR in collections 1 and 2. These images were acquired at different sites across the United States and Canada. ADNI1: The images from the Alzheimer’s Disease Neuroimaging Initiative (ADNI) database (http://adni.loni.usc.edu) used in this study consist of 228 control subjects from the 1.5T baseline collection. These images were acquired on 1.5T MR scanners at 60 different sites across the United States and Canada. A standardized MRI protocol to ensure cross-site comparability was used. Typical MR images are 3D sagittal MPRAGE (TR = 2,400 ms, minimum full TE, TI = 1,000 ms, flip angle: 8º, 24 cm FoV, and a 192 x 192 x 166 acquisition matrix in the x-, y-, and z- dimensions, yielding a voxel size of 1.25 x 1.25 x 1.2 mm^3^, later reconstructed to get 1 mm^3^ isotropic voxel resolution). ADNI2: The images from the ADNI2 database (second phase of the ADNI project) consist of 215 control subjects. Images were acquired on 3T MR scanners with the standardized ADNI-2 protocol, available online (www.loni.usc.edu). Typical MR images are T1-weighted 3D MPRAGE sequence (TR = 2,300 ms, TE = 2.98 ms, flip angle 9º, FoV = 256 mm, resolution 1.1 x 1.1 x 1.2 mm^3^).
  4. ICBM (N = 293): The images from the International Consortium for Brain Mapping (ICBM) dataset (http://www.loni.usc.edu/ICBM/) were obtained on 293 subjects through the LONI website. The MR images are T1-weighted MPRAGE (fast field echo, TR = 17 ms, TE = 10 ms, flip angle = 30º, 256 x 256 matrix, 1 mm^2^ in plane resolution, 1 mm thick slices) acquired on a 1.5T Philips GyroScan imaging system (Philips Medical Systems, Best, The Netherlands).
  5. ABIDE (N = 256): The images from the Autism Brain Imaging Data Exchange (ABIDE) dataset (http://fcon_1000.projects.nitrc.org/indi/abide/) used in this study consist of 256 subjects acquired at 20 different sites on 3T scanners.

The datasets HCP, IXI, and OpenNeuro were used for supervised training for in-domain test. The Bordeaux and OASIS datasets were used for out-of-domain test. Finally, the Lifespan dataset was used for semi-supervised training. Table 1 summarizes the used datasets.

**Table 1.** Summary of the six datasets used throughout the study.

***DATASET***	HCP (T1&T2)*	IXI (T1&T2)*	OpenNeuro (T1&T2)*	***Bordeaux (T1&T2)^†^***	***OASIS (T1)^†^***	**Lifespan** ***(T1)^‡^***
*Train*	*1,089*	*414*	*258*			*3,067*
*Test*	*10*	*3*	*7*	*44*	*100*	
*Val*	*5*	*3*	*4*			

HCP, IXI, and OpenNeuro, which makes up the train dataset, are indicated by (*). Bordeaux and OASIS are only used for testing and are indicated by (†), and Lifespan for the semi-supervised training approach are indicated by (‡).

### 2.2 Preprocessing

The preprocessing stage is essential to locate the images into a standardized geometric and intensity space which simplifies the learning process.

Firstly, all the images (T1 and T2) were denoised using a spatially adaptive non-local means filter (Manjón et al., 2010). Then, for the standardization of geometric spaces, a rigid registration parameter estimation between T1 and T2 images (T2 was registered to the space of the same subject T1) was performed. Later, an affine registration of the T1 image to the MNI152 space at 1 ${\text{mm}}^{3}$ using the ANTS software (Avants et al., 2009) was done. Finally, the T2 image was also registered to the MNI152 space by concatenating the rigid (T2 to T1) and affine T1-based transformations. Afterward, we performed an inhomogeneity correction of the registered T1 and T2 images using the N4 method (Tustison et al., 2010).

Finally, we standardized the intensity levels across our data. It is crucial to recognize that generating a T2 image from a T1 image requires learning and applying a specific intensity transformation. Therefore, normalizing intensities during preprocessing is essential. This step aligns the intensity distributions between the source and target images, thereby reducing complications arising from the diverse acquisition protocols of T1 (MPRAGE, SPGR, etc.) and T2 images (a many-to-many mapping problem). By implementing contrast stretching techniques during preprocessing, we ensure that both sets of images share similar intensity properties, ultimately facilitating the synthesis process. This fact has already been studied in Reinhold et al. (2019) where they examined the importance of image normalization as an essential step in preprocessing, analyzing seven normalization algorithms. Their findings concluded that normalization is crucial for accurate MRI synthesis.

In our case, two techniques for intensity normalization were used. For T1 images, we used an approach based on Trimmed Means Segmentation (TMS) (Manjón et al., 2008) that adjusts the histogram using a piece-wise linear function. The method uses the mean values of the different brain tissues for matching. For T2 images, we used a modified version of histogram matching (Gonzales & Fittes, 1977). Histogram matching has been widely used to normalize image intensities. However, when the origin and target images have very different tissue proportions (for example, small and large ventricles), it can produce incoherent results. To minimize this problem, before applying histogram matching, we non-linearly register the reference image to the target image to minimize these shape variations using a deep learning-based non-linear registration network (Coupé et al., 2020). Finally, both T1 and T2 images were renormalized by dividing each by its mean value, mapping them into a range better suited for neural network training.

### 2.3 Neural network architecture

In medical image synthesis tasks, the U-Net architecture (Ronneberger et al., 2015) is a popular choice. However, when dealing with entire 3D images, the memory requirements are high, which limits the number of filters the network can use. This, in turn, restricts its effectiveness in more complex scenarios. As a result, most synthesis methods are based on 2D slices to overcome these limitations.

**Fig. 1.**
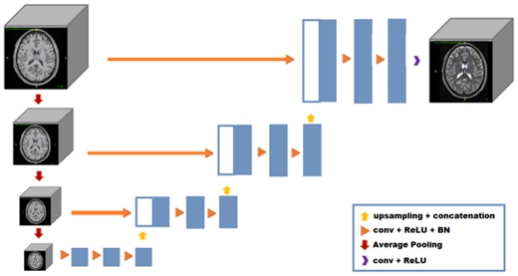
DPN synthesis network architecture. First the 3D average pooling layers are used to lower the input resolution at different scales and then the convolution layers at each scale are applied.

In this work, we propose to use an alternative architecture, the Deep Pyramidal Network (DPN) network (Morell-Ortega et al., 2025). Due to its reduced complexity and fewer parameters, the DPN requires less computational power and shorter training times while potentially maintaining comparable or superior performance in image synthesis. The DPN was first proposed for segmentation purposes (Morell-Ortega et al., 2025). This architecture is based on an incremental coarse-to-fine approach which enables a lighter and less memory-demanding model. Compared with a U-Net where an encoder–decoder strategy is used, the DPN consists only of the decoder part. The network starts with an input tensor, the original high-resolution volume, and progressively generates lower-resolution inputs at 1/2, 1/4, and 1/8 scales using a 3D Average Pooling layer. At the deepest level, features are extracted through three processing blocks (Convolution + ReLU + Batch Normalization). These features are then up-sampled using trilinear interpolation resizing and merged with higher-resolution features from the previous level to refine details. This process continues until the original resolution is restored, where a final Convolution + ReLU layer generates the output intensities. Each convolution is performed with a kernel of size 3 x 3 x 3, using the same number of filters in all blocks. In this project, we used 32 filters at each convolution, which results in a DPN network with a total of learnable 395.169 parameters. The general idea of this architecture is to use an incremental approach where the first layers generate the most general patterns that are progressively refined as the network increases its resolution (see Fig. 1 for details).

### 2.4 Atlas generation

One of the primary challenges faced by deep learning-based methods is their sensitivity to images that differ from those used during training (i.e., out-of-domain generalization). Consequently, performance can decline significantly when these models are applied to different datasets. The goal of our proposed method is to ensure robustness across various image patterns and anatomical variations, thereby minimizing the risk of catastrophic errors. To achieve this, we propose incorporating prior information in the form of a T2 atlas to support the synthesis task. Although atlas-based methods may not achieve the peak accuracy of deep learning approaches, they are considerably more robust when handling diverse image conditions. Leveraging this *a priori* information can reduce model sensitivity to noise, artifacts, or variability in the input data, thus preventing catastrophic errors and implausible topologies.

We employ a multi-atlas strategy to create a subject-specific atlas, which has proven more robust than a single-atlas approach (Coupé et al., 2011). Specifically, the method estimates a nonlinear registration transform from a subset of anatomical T1 volumes in the training library, aligning each to the input T1 volume. These nonlinear transforms are then applied to the corresponding T2 volumes in the library, resulting in a subject-specific set of T2 volumes. Finally, these warped T2 volumes are merged voxel-wise according to a rule that assigns an intensity value to each voxel.

To perform the intensity fusion, an intensity-based weighted majority voting is used where voxels of the library with intensities similar to the voxel to be processed have a higher weight than those more dissimilar. Each weight is calculated as follows:

$$w_{p}=\frac{1}{1+d\;\cdot \;\left\|\;I_{p}-\;L_{p,j}\;\right\|\;},$$ (1)

where *d* is a scaling coefficient, *$I_{p}$* is the intensity of a voxel at a position *p*, and $L_{p,j}$ is the intensity of a voxel at a position *p* of an image *j* from library. We set *d* as 0.5 experimentally. To deal with the temporal limitations of multi-atlas approaches, the fusion algorithm was implemented in C language and parallelized using the OpenMP multiprocessing library.

Nonetheless, the primary limitation of this methodology, as previously identified in the article by Park et al. (2014), is the time cost associated with non-linear registrations and intensity fusion. Fortunately, deep learning-based non-linear registration methods have dramatically reduced the time cost of non-linear registration from minutes to less than 1 second (Balakrishnan et al., 2019). In the proposed method, we used a deep non-linear registration network developed in Coupé et al. (2020).

**Fig. 2.**
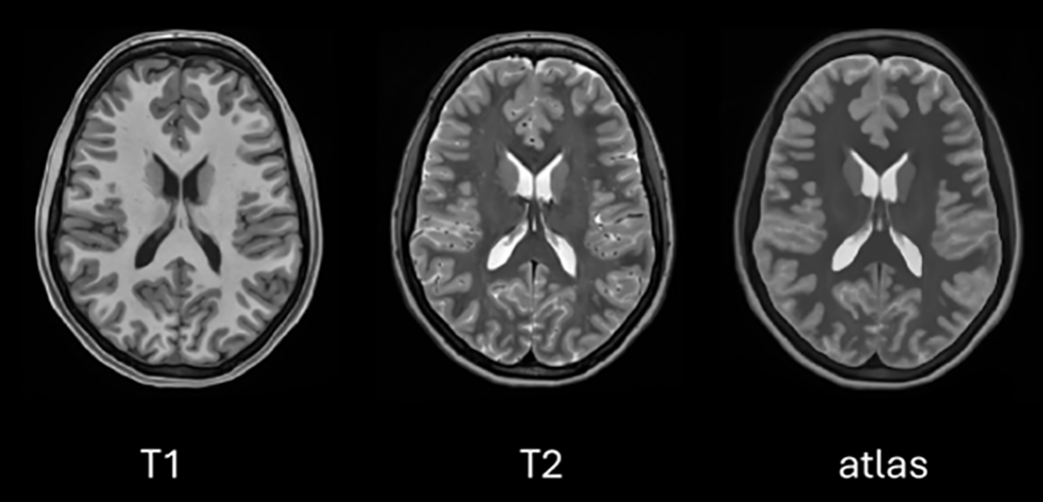
Example of an atlas obtained for an input T1 case. From left to right: original T1 image, its corresponding T2 image, and atlas obtained for the input T1 image. Note that although the fine details are not visible in the atlas, the overall appearance is well captured by the atlas.

Another aspect that impacts the temporal cost of the atlas generation process is the number of templates used to generate it (the bigger the library, the more time will be spent on the atlas creation). We used a subset of 70 HCP T1/T2 volumes but selected only the N cases that are more similar to the sample under analysis (based on the L1 norm of image intensities) to perform the non-linear registration and the later fusion (T1 and T2 images were already intensity normalized). We empirically found that a plateau in terms of image quality is reached when the size of the library is N = 20 samples (see Fig. 2 for an example of a generated atlas). Consequently, the atlas creation process takes around 20 seconds on our hardware configuration. This atlas is normalized by its mean and integrated into the architecture alongside the input T1 image as an additional input channel.

### 2.5 Training

Selecting an appropriate loss function is crucial when training a neural network. To determine the most effective option for T2 synthesis, we evaluated several loss functions. Among the options tested, the mean absolute error (MAE) is commonly used in most synthesis studies (Chartsias et al., 2018; S. Pan et al., 2023; Zhu et al., 2023) because it directly measures the average difference in pixel intensity between the generated and real images, which is particularly useful in tasks where maintaining accuracy in pixel intensity is crucial. However, this loss tends to produce blurred images, failing to recover fine details (Chen et al., 2023).

Another widely used loss function is the adversarial loss, typically employed in GAN-based methods. In these approaches, a discriminator assesses the plausibility of generated samples by distinguishing them from real images, which in turn guides the generator to produce more realistic outputs. However, training GANs can be unstable due to challenges in balancing the generator and discriminator (Upadhyay & Awate, 2021). This instability often results from the delicate equilibrium required between the generator and discriminator. Consequently, the quality of generated images can vary significantly, with some outputs appearing highly realistic while others exhibit artifacts or hallucinated anatomical structures.

In contrast, perceptual loss takes a different approach to evaluate the quality of the generated sample by focusing on higher-level image features rather than focusing solely on pixel-level differences or image plausibility. It operates by comparing the feature activations of a pre-trained deep network between the generated image and the real image (a common network used for this task is the VGG (Dar et al., 2019; Johnson et al., 2016) for 2D). These networks are trained on large datasets to recognize high-level image features critical for perceptual quality. By aligning the features in the latent space, perceptual loss helps ensure that the generated images resemble the real ones visually and share their underlying structural and semantic information.

While perceptual loss functions, such as those utilizing the VGG network, have effectively enhanced image quality by focusing on high-level features, their applicability to medical imaging, particularly MRI neuroimaging, is limited. On the one hand, VGG typically operates in 2D, while our data are in 3D. On the other hand, models such as VGG are trained on natural images and may not capture the unique characteristics of medical images. For instance, M. Han et al. (2022) and Mei et al. (2022) highlight that using perceptual loss with networks trained on natural images may not be suitable for medical imaging tasks due to the distinct nature of medical images. To address this, employing the encoder of a self-trained autoencoder using MR data allows for extracting features more pertinent to the specific problem, thereby improving the relevance and effectiveness of the perceptual loss in the synthesis task.

However, despite its advantages, perceptual loss has some practical challenges. Training a synthesis deep network with perceptual loss, especially in 3D, is computationally and memory intensive, as it requires processing high-dimensional data at both networks (generator and latent space of the autoencoder). This limitation motivates the exploration of alternative loss functions, such as those based on the frequency domain.

Analyzing signals in the frequency spectrum is particularly relevant for MRI images, as these images were originally acquired in this domain. In theory, frequency components provide truly global indirect anatomical information compared with perceptual loss where the visual field depends on the depth of the autoencoder. The Fourier Transform enables analyzing and manipulating an image’s frequency content. In this domain, each point represents information from the entire spatial domain, capturing global image characteristics. This duality between spatial and frequency domains allows for the development of loss functions that emphasize global features, potentially improving the quality of synthesized images while serving as an efficient alternative to perceptual loss. Additionally, operations in the frequency domain are computationally efficient in most programming frameworks.

This type of loss function is not new, and it has been previously used in super-resolution problems where the objective is to recover the high-frequency content of the image (Fuoli et al., 2021). In image synthesis, it has also been recently used. Jiang et al. (2021) introduced Focal Frequency Loss (FFL), a novel loss function designed to enhance image reconstruction and synthesis by focusing on hard-to-learn frequency components. In this work, we propose an FFT-based loss computed as the mean squared error between the complex spectra of the predicted and ground truth samples.

Finally, we would like to prioritize also the practical applications of the network under design, alongside its reconstruction quality. We believe this balanced approach will enhance its overall effectiveness. For example, GANs or diffusion models can create images with impressive details. However, this realism is not always linked to probed clinical utility. Dohmen et al. (2024) examine how visual improvement in medical image synthesis is a subjective aspect that is challenging to quantify accurately. Therefore, considering that one of the goals of synthesizing T2 images from T1 images is to provide more information to improve segmentation, we propose to include a segmentation-based loss in the learning process. This approach aims to synthesize a T2 image that is good in terms of image metrics but also generates a good segmentation. Measuring the Dice coefficient as an auxiliary evaluation metric informs us not only about the image’s visual quality but also about its ability to produce an improved segmentation.

**Fig. 3.**
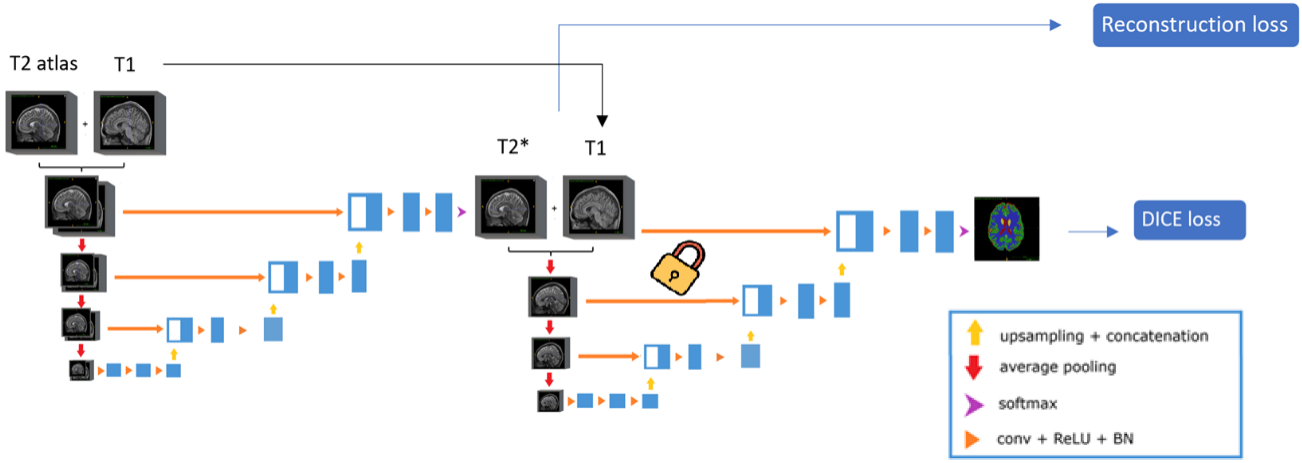
DPN synthesis network with Dice loss included in the learning process by adding the frozen segmentation network. The synthesis network receives T1 and T2 atlas as input. The segmentation network receives the same T1 and the synthetic T2 (T2*) generated by the synthesis network. The reconstruction loss consists of an image fidelity term.

To incorporate this approach, we first trained a multi-modal (T1 + T2) brain tissue segmentation network capable of segmenting seven brain regions (cerebral-spinal fluid, white matter, cortical gray matter, subcortical gray matter, cerebellar gray matter, cerebellar white matter, and brainstem). We decided to train a multimodal network (T1 + T2) instead of a monomodal (T2) to promote the consistency between the synthesized T2 and the real T1. The network was trained using 70 paired T1 and T2 images from the holiAtlas dataset (Manjón et al., 2025). Due to memory constraints, we utilized a compact DPN architecture with only 8 filters per level, resulting in a total of 87.674 parameters. Once trained, the segmentation network was frozen and integrated into the synthesis pipeline to compute the loss function (see Fig. 3 for details).

### 2.6 Semi-supervised learning

One of the primary objectives of this work is to develop a robust T2 synthesis model from T1 images, ensuring generalizability across diverse datasets and clinical scenarios (for instance, highly atrophic brain images or containing lesions). A significant challenge in achieving this goal is the limited availability of large enough datasets containing paired T1 and T2 images, which is essential for traditional supervised learning approaches. To address this limitation, we employed a semi-supervised strategy that enables the integration of unpaired data, significantly increasing both the diversity and volume of training samples.

To this end, we make use of the Lifespan dataset, a large collection of 3,067 T1 images from male and female participants aged 3 to 90 years. This dataset provides a rich anatomical and demographic diversity (including pathological images) but lacks paired T2 images. To incorporate this dataset into training, we generated synthetic T2 images using the best previously trained supervised model. These synthetic T2 images act as pseudo-images, allowing us to fine-tune the synthesis network with a broader set of anatomical variations. This approach is analogous to pseudo-labeling techniques commonly used in semi-supervised segmentation (Han et al., 2024; Kamraoui et al., 2022).

**Fig. 4.**
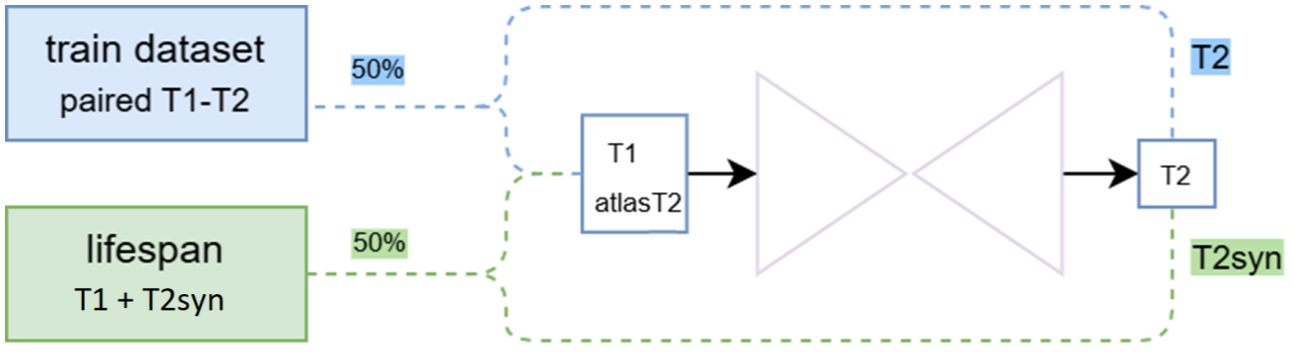
Schematic of the semi-supervised approach: 50% of the time, the model reads from the train dataset with real T2 as ground truth, and 50% of the time it reads from the Lifespan dataset with T2 synthesized with a previously trained model used as ground truth.

By integrating real paired and pseudo-synthetized paired data, our method benefits from an expanded training distribution, enabling better generalization to unseen cases. In particular, this strategy ensures that the model is exposed to a wide range of brain ages (or brain shapes), varying acquisition conditions, and potential pathologies such as atrophy and microvascular lesions, which are often underrepresented in limited paired datasets. The segmentation labels required for the loss function on the Lifespan dataset were obtained using a monomodal segmentation network (using 32 filters) trained only with T1 images from the holiAtlas dataset (the improved quality of the T1 segmentations forced the network to learn better mappings despite the limitation of using pseudo-images). To maintain a balance between real and semi-supervised data, training samples were selected with a 50% probability, preventing overfitting to synthetic cases while still leveraging the increased data diversity. The process is schematically illustrated in Figure 4.

All the experiments were performed with Tensorflow 1.15, using a V100-SXM2-32GB GPU with a system with 64GB RAM memory and an Intel Xeon processor Gold 5220R CPU @ 2.20GHz with 32 cores, all running under Linux Ubuntu 18.04.5. All networks were trained with 20 steps per epoch and a batch size of 1 for 1,500 epochs (1 day of processing), ensuring that their convergence was reached. Adam optimizer (Kingma & Ba, 2014) was used with its default parameters.

## 3 Results

In this section, we summarize the results of the experiments performed to evaluate the proposed approach. First, we present the results of the supervised model with the optimal combination of loss functions and the results of the semi-supervised learning. Finally, a comparison with some methods of the state of the art is included.

Statistical differences were tested using the two-sided Wilcoxon signed-rank test (p < 0.05). Because multiple comparisons were performed, a correction was applied to control the false discovery rate (FDR) using the Benjamini–Hochberg method (Benjamini & Hochberg, 1995). The original p-values for each comparison were calculated and adjusted using the FDR correction.

### 3.1 Supervised learning results

The proposed synthesis network was first trained with the supervised dataset composed of paired T1 and T2 images from the HCP, OpenNeuro, and IXI datasets. The metrics used to evaluate the results were the Peak Signal to Noise Ratio (PSNR), the Pearson’s Correlation Coefficient (CC), the Structural Similarity Index Measure (SSIM) (Wang et al., 2004), and the Dice coefficient. All metrics were estimated inside the intracranial cavity, extracted from the T1 images using Deep ICE method (Manjón et al., 2020). The results of different analysis following this supervised paradigm are shown in Tables 2, 3, and 4.

**Table 2.** Results for in-domain test data (N = 20).

	PSNR	CC	SSIM	Dice
MAE(space domain)+ $\lambda_{1}\;\cdot \;MSE\left(FFT\right)+\;\lambda_{2}DiceLoss+atlas$	**32.1432** **±** 1.4662*	**0.9865** **±** 0.0052*	**0.9603** **±** 0.0100*	**0.9082** **±** 0.0982*
MAE(space domain)+ $\lambda_{1}\;\cdot \;MSE\left(FFT\right)+\;\lambda_{2}DiceLoss$	31.8454 ± 1.3386	0.9855 ± 0.0050	0.9582 ± 0.0089	0.9012 ± 0.1023
MAE(space domain)+ $\lambda_{1}\;\cdot \;MSE\left(FFT\right)$	31.8702 ± 1.2455	0.9856 ± 0.0050	0.9570 ± 0.0089	0.8813 ± 0.1299
MAE(space domain)+ $\lambda_{1}\;\cdot \;MAE\left(FFT\right)+$	27.6085 ± 0.9549	0.9649 ± 0.0060	0.9098 ± 0.0125	0.7879 ± 0.2406
MAE(space domain)+ $\lambda_{3}\;\cdot \;Perceptual$	31.4378 ± 1.6187	0.9849 ± 0.0060	0.9577 ± 0.0106	0.8861 ± 0.1254
MAE(space domain)	24.9342 ± 0.7783	0.9306 ± 0.0164	0.8518 ± 0.0135	0.7310 ± 0.2857

Best results in bold.

* Represents statistically significant differences between the first and second row (the best model and the second best).

**Table 3.** Results for out-of-domain Bordeaux dataset test data (N = 44).

CONFIGURATION	PSNR	CC	SSIM	Dice
MAE(space domain)+ $\lambda_{1}\;\cdot \;MSE\left(FFT\right)+\;\lambda_{2}DiceLoss+atlas$	**28.4298** **±** 0.7679*	**0.9712** **±** 0.0043*	**0.9143** **±** 0.0118*	0.8543 ± 0.0306
MAE(space domain)+ $\lambda_{1}\;\cdot \;MSE\left(FFT\right)+\;\lambda_{2}DiceLoss$	27.4602 ± 0.9670	0.9627 ± 0.0077	0.9091 ± 0.0133	**0.8599** **±** **0.1494**
MAE(space domain)+ $\lambda_{1}\;\cdot \;MSE\left(FFT\right)$	27.4417 ± 0.9625	0.9625 ± 0.0067	0.9106 ± 0.0123	0.8314 ± 0.1954
MAE(space domain)+ $\lambda_{1}\;\cdot \;MAE\left(FFT\right)+\;$	27.2069 ± 1.0471	0.9603 ± 0.0089	0.8864 ± 0.0115	0.7865 ± 0.2509
MAE(space domain)+ $\lambda_{3}\;\cdot \;Perceptual$	26.7039 ± 0.8444	0.9580 ± 0.0074	0.9043 ± 0.0130	0.8307 ± 0.1935
MAE(space domain)	25.2364 ± 1.0470	0.9390 ± 0.0160	0.8343 ± 0.0159	0.7408 ± 0.2890

Best results in bold.

* Represents statistically significant differences between the best and second best (the best model and the second best).

As discussed in Section 2.5, we have experimented with diverse loss functions. In Table 2 we studied the impact of each of them carefully through an ablation study. The results are analyzed over the in-domain dataset, letting apart the out-of-domain one for an independent analysis. In the image space, we used as baseline loss the Mean Absolute Error (MAE). As W. Li et al. (2020) demonstrated, using L1 loss encourages less blurring compared with L2 loss. However, in the frequency domain, different studies use different L-norms, the L1 (MAE) in Fuoli et al. (2021) and L2 (MSE) in Jiang et al. (2021). Therefore, we experimented with both of them to assess their suitability. The final loss used for training is a mix of several balanced losses.

$$\begin{array}{l} L_{Total}=MAE\left(space\;domain\right)+\;\lambda_{1}\cdot MSE\left(FFT\right)\\ \;\;\;\;\;\;\;\;\;\;\;\;\;\;\;+\;\lambda_{2}DiceLoss. \end{array}$$ (2)

The values of the different λ  were set so that all loss terms have the same magnitude, in order to help maintain a balanced optimization process. The values are $\lambda_{1}=0.01$ and $\lambda_{2}=150$ . The experiments in Table 2 demonstrate that *using* the MAE of the FFT instead of the MSE of the FFT results in lower performance metrics, indicating that MSE is more effective for this task. Furthermore, when the FFT computation is removed entirely, leaving only the MAE between the real and predicted images, the metrics drop significantly, suggesting that MAE alone is insufficient for the synthesis task. Additionally, analyzing the impact of perceptual loss, we observed that it achieves comparable results to the MSE of the FFT for in-domain testing in terms of reconstruction metrics, corroborating our assumption that FFT can work as an efficient alternative to perceptual loss.

**Fig. 5.**
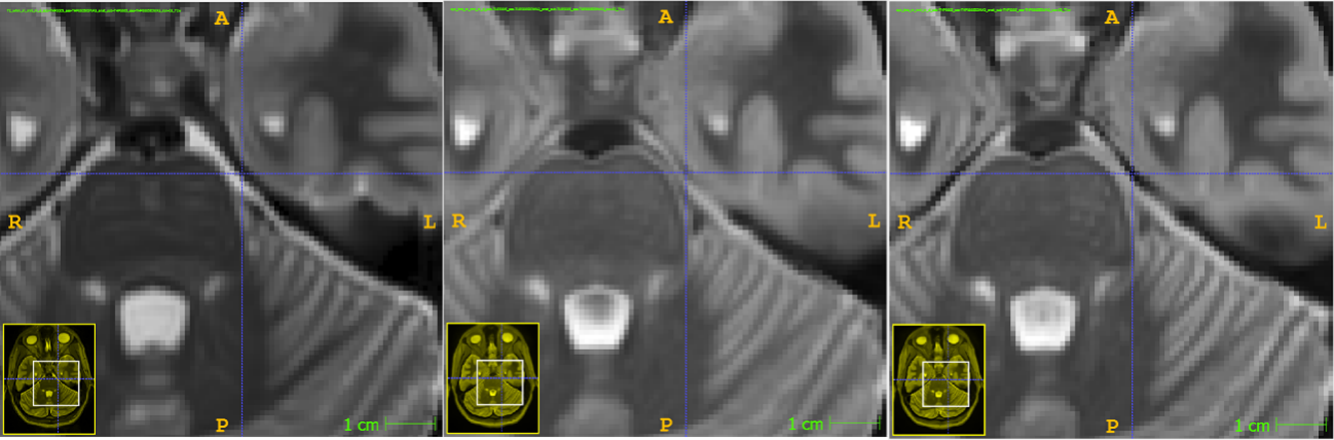
On the left is real T2, in the middle is synthetic T2 with the method without segmentation loss, on the right is synthetic T2 with the method with segmentation loss. The model without segmentation loss does not clearly delimit the area between the cerebrospinal fluid and the cerebellum or the brainstem.

The impact of segmentation loss was also analyzed in Table 2. Incorporating it into the generator’s loss function quantitatively improves the Dice metric, as it encourages the preservation of segmentation-relevant structures in the synthesized images. Qualitatively, as shown in Figure 5, the absence of Dice loss leads to blurrier edges, particularly in small structures such as arteries and cerebrospinal fluid boundaries. Though small, these structures enhance both the image’s perceived quality and its usefulness for downstream tasks. Segmentation loss acts as a focal mechanism, directing attention toward regions with lower Dice scores and improving their synthesis. Without it, the model struggles to generate these fine details accurately.

Furthermore, we observed that incorporating the atlas significantly improves both reconstruction and segmentation metrics statistically. As we will analyze later, the atlas provides the network with *a prior* that helps it learn the synthesis task more effectively—rather than starting from scratch—and enhances its robustness when handling input data that differ significantly from the training distribution.

In Table 3, we consider the results on the Bordeaux dataset, which serves as an out-of-domain evaluation. We aim to assess the model’s ability to generalize to data from different sources, characterized by greater heterogeneity and, in some cases, the presence of lesions. On this dataset, we again observed the benefits of complementing MAE in image space with additional loss functions. Notably, the trend between MAE and MSE in the frequency domain remains consistent, confirming that MSE is the superior choice, and its performance is comparable with perceptual loss. Additionally, segmentation loss improves the Dice score and contributes to better overall reconstruction quality, as reflected in the reconstruction metrics.

**Fig. 6.**
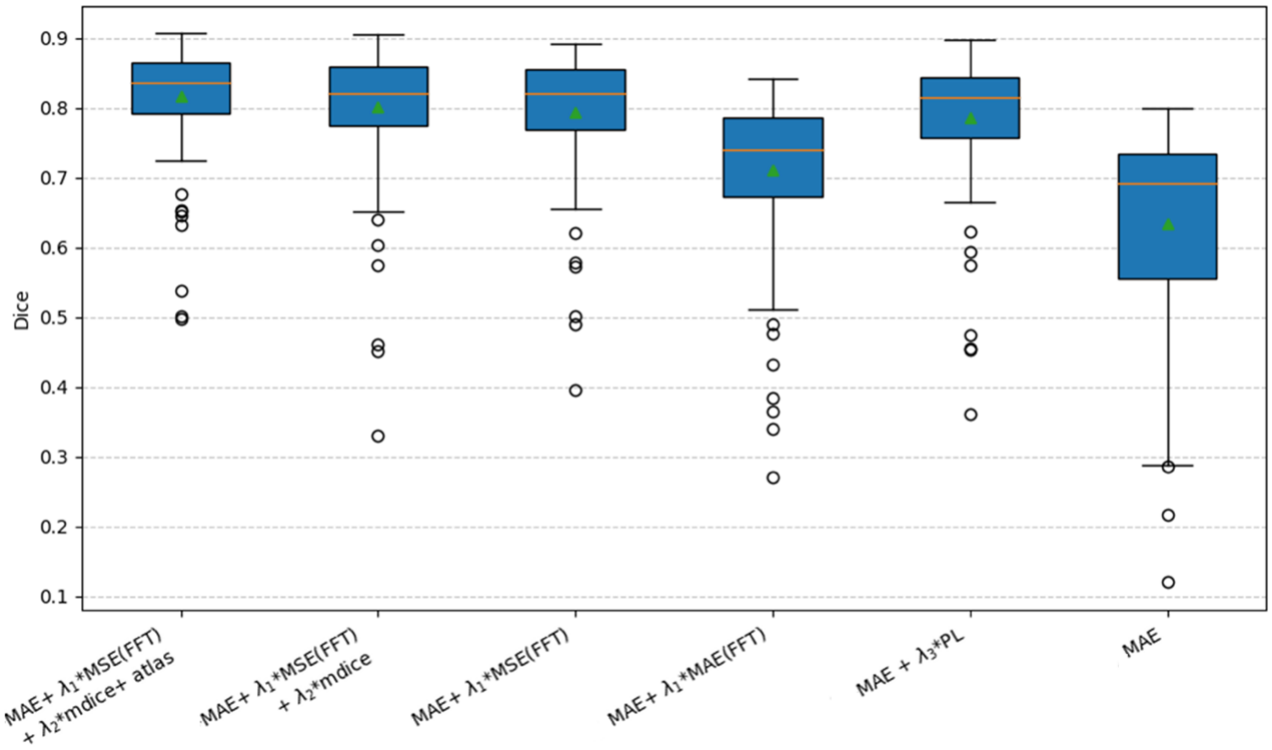
Dice distribution by model in OASIS dataset. Measuring the difference between the segmentation obtained with the unimodal segmentation network with a T1, and the segmentation obtained with the multimodal network, with the T1 and the T2 synthesized for each method. Indirect measure of the quality of the synthesized T2.

Finally, we analyze the results from the second out-of-domain dataset from OASIS. In this case, we do not have a T2 image to compare the reconstruction quality. However, we can indirectly assess the quality of the generated volume using the segmentation metrics as a proxy. As shown in Table 4, the same trend observed in the in-domain test dataset evaluation is repeated here. On one hand, the equivalence between perceptual loss and MSE in the spectrum is confirmed. On the other hand, the introduction of segmentation loss makes the synthesized images easier to segment, impacting in the measured DICE. Finally, it is revalidated that incorporating prior information in the form of an atlas is beneficial. Figure 6 shows data distribution to better understand the results.

**Table 4.** Dice results in OASIS dataset (N = 100).

CONFIGURATION	Dice
MAE(space domain)+ $\lambda_{1}\;\cdot \;MSE\left(FFT\right)+\;\lambda_{2}DiceLoss+atlas$	0.8158* **±** 0.0776*
MAE(space domain)+ $\lambda_{1}\;\cdot \;MSE\left(FFT\right)+\;\lambda_{2}DiceLoss$	0.8017 ± 0.0936
MAE(space domain)+ $\lambda_{1}\;\cdot \;MSE\left(FFT\right)$	0.7942 ± 0.0884
MAE(space domain)+ $\lambda_{1}\;\cdot \;MAE\left(FFT\right)+\;$	0.7106 ± 0.1114
MAE(space domain)+ $\lambda_{3}\;\cdot \;Perceptual$	0.7859 ± 0.0950
MAE(space domain)	0.6346 ± 0.1444

Measuring the difference between the segmentation obtained with the unimodal segmentation network with a T1, and the segmentation obtained with the multimodal network, with the T1 and the T2 synthesized for each method. Indirect measure of the quality of the synthesized T2. Best results in bold.

* Represents statistically significant differences between the first and second row (the best model and the second best).

### 3.2 Atlas impact

Previous studies have employed atlases as prior information to enhance segmentation quality by providing contextual localization data (Morell-Ortega et al., 2025). It is well established that atlas‐based approaches leverage spatial information through registration, resulting in greater consistency and interpretability. In our approach, we integrate the atlas as an input channel in a 3D CNN deep learning network to help ensure that the network’s predictions remain spatially consistent. The use of the multi-atlas-based synthesized image serves as a starting point for the network.

In order to further measure whether substantial improvements in robustness and consistency justify the extra cost of using atlases, we performed an additional quantitative and qualitative analysis. Our quantitative evaluation used the out-of-domain test dataset from Bordeaux with data transformations applied via the TorchIO library (Pérez-García et al., 2021), which serves us to measure the model’s ability to generalize under varying input conditions.

We applied realistic transformations that simulate clinical acquisition conditions, where image quality may vary compared with research datasets. These transformations, applied with random degrees, include anisotropy, blurring, gamma correction, bias field, motion artifacts, noise, and ghosting. Given that the network’s primary objective is contrast synthesis rather than correction of spatial acquisition parameters, T2 images are also subjected to anisotropy and blurring to reflect their naturally lower resolution under such conditions. For the rest of the transformations, modifications are applied solely to the T1 input volume (from which the atlas is generated again), while the T2 original target image remains unchanged. The atlas is recalculated for these transformations based on the augmented T1 input, thereby simulating the network’s response under real-world conditions. This approach allows us to categorize the transformations into geometric and intensity-based groups, facilitating a more rigorous analysis of the model’s performance. TorchIO augmentations were produced using the default parameters with the exception of flip (done in the three axes) and log_gamma (range = [-0.1, 0.1]).

**Fig. 7.**
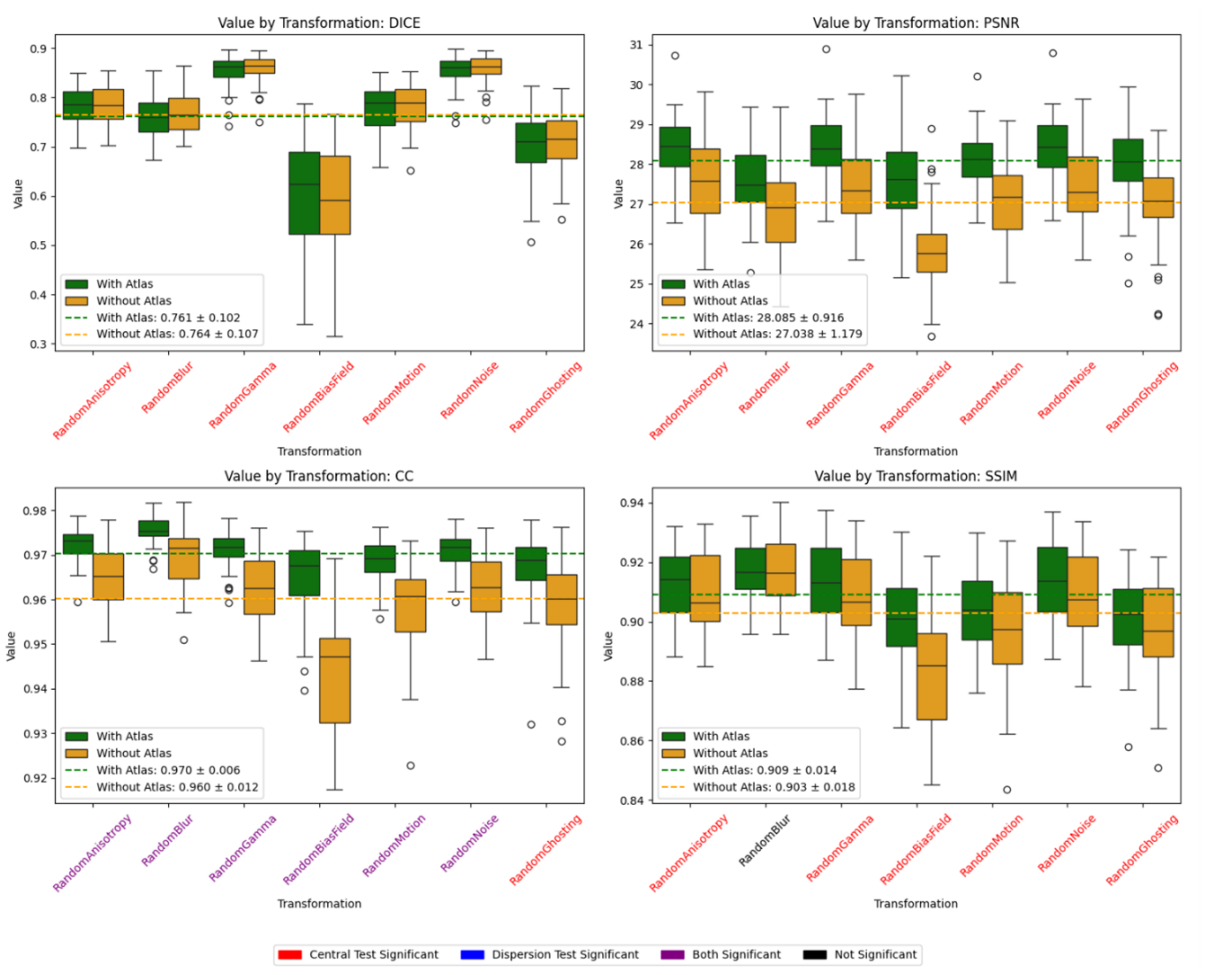
Impact of Atlas Incorporation on image synthesis quality across different transformations over Bordeaux dataset. Dotted lines are the mean for all the transformations.

Figure 7 presents a comparative analysis of different image quality metrics (DICE, PSNR, CC, and SSIM) across multiple transformations, evaluating the effect of incorporating an atlas. The results indicate that models using an atlas achieve higher mean values across all metrics, except DICE, while reducing dispersion in some transformations, suggesting improved robustness to perturbations. Statistical significance is assessed through central tendency and dispersion tests. For dispersion analysis, if both distributions are normal (Shapiro–Wilk test), Bartlett’s test is applied; otherwise, Levene’s test accounts for non-normal distributions. To mitigate the risk of false positives due to multiple comparisons, a Bonferroni correction is applied, adjusting the significance threshold accordingly. Cases where significance is found in central tendency, dispersion, or both tests are highlighted in red, blue, and purple, respectively. Notably, bias field, motion, and gamma transformations exhibit significant differences, with models without an atlas experiencing greater performance degradation. While some transformations show marginal differences, the overall trend highlights the benefit of integrating an atlas, particularly in maintaining structural consistency (DICE) and correlation (CC).

**Fig. 8.**
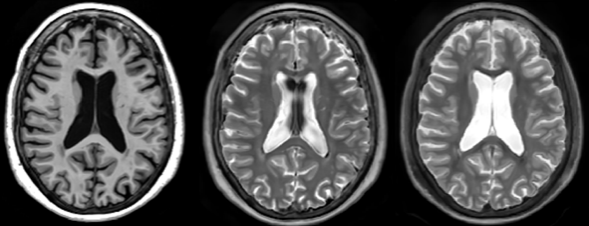
From left to right: T1 input, T2 image synthesized with the synthesis model without the atlas as a channel and image obtained with the synthesis model with atlas. Note that lateral ventricles are correctly synthetized with the atlas option but not without it.

Visually, the effects of the atlas can also be compared in Figure 8, where an example of the synthetic T2 obtained with the atlas method and without the atlas is shown. The model without atlas makes serious mistakes in the cerebrospinal fluid of the ventricles.

To the best of our knowledge, this is the first work that directly integrates atlas priors as an input channel into a 3D CNN for the specific task of T2 synthesis from T1 images. While previous approaches have leveraged atlas‐based registration or deep learning techniques separately, our unified framework combines these strategies providing both enhanced contextual awareness and improved robustness.

### 3.3 Semi-supervised learning results

The semi-supervised approach was designed to enhance the robustness and generalization capabilities of the proposed method, enabling it to perform effectively on a large variety of unseen data. Incorporating unpaired images alongside paired data into the training process, the model leverages a more extensive and diverse dataset, capturing broader variations in anatomical structures and imaging protocols. The results of the semi-supervised training are shown in Table 5.

**Table 5.** Semi-supervised and supervised training comparison (for in-domain (N = 20) and out-of-domain (N = 44)).

		PSNR	CC	SSIM	Dice
In-domain	Semi-supervised	**32.2718** **±** 1.4842*	**0.9869** **±** 0.0050*	**0.9612** **±** 0.0097*	**0.9095** **±** **0.0975**
	Supervised	32.1432 ± 1.4662	0.9865 ± 0.0052	0.9603 ± 0.0100	0.9082 ± 0.0982
Out-of-domain (Bordeaux)	Semi-supervised	28.3450 ± 0.7679	0.9712 ± 0.0043	**0.9152** **±** 0.0122*	**0.8583** **±** 0.0275*
	Supervised	**28.4298** **±** 0.7679*	**0.9717** **±** 0.0043*	0.9143 ± 0.0118	0.8543 ± 0.0306

Best results in bold.

* Represents statistically significant differences between semi-supervised and supervised training.

On the in-domain dataset, both methods perform nearly identically across all metrics, suggesting that when test images closely match the training distribution, each approach effectively captures the underlying image characteristics. For the out-of-domain (Bordeaux) data, the semi-supervised method achieved statistically significant improvements in SSIM and Dice score. Although these differences are modest in magnitude, the statistical significance suggests that the semi-supervised approach might better preserve structural details and segmentation quality when encountering data that differ from the training set. Nonetheless, the practical implications of these improvements should be interpreted with caution.

**Fig. 9.**
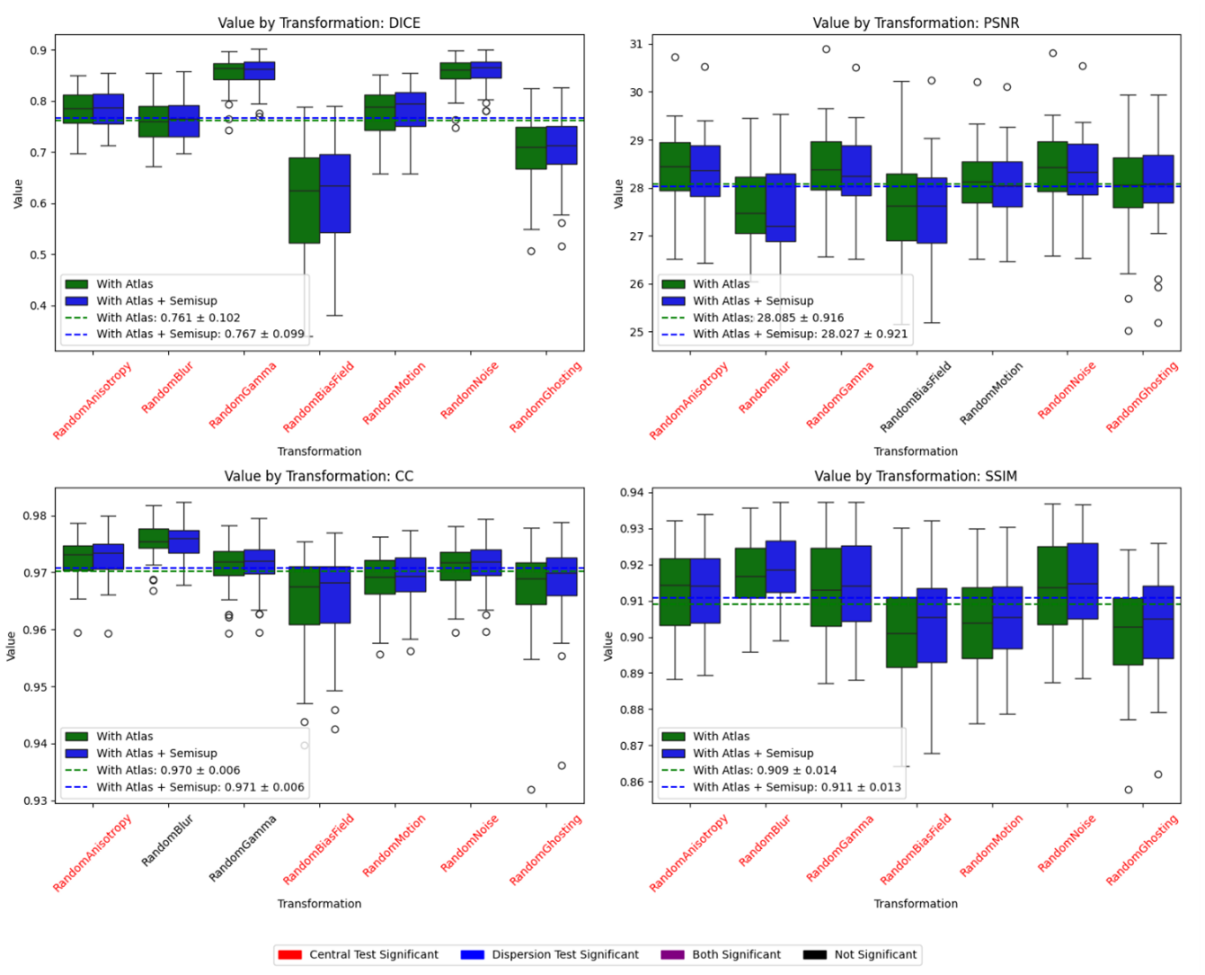
Impact of semi-supervised training on image synthesis quality across different transformations. Dotted lines are the mean for all the transformations.

However, when we repeated the analysis by applying data augmentation to the Bordeaux test dataset (see Fig. 9), we observed that this approach once again enhances the model’s robustness, achieving higher average values for Dice, Correlation Coefficient, and SSIM. In summary, atlas provides prior anatomical information, while semi-supervised training leverages unlabeled or partially labeled data to learn robust features. Combined, they enhance both image quality (PSNR, SSIM, CC) and segmentation accuracy (Dice), especially under transformations meant to simulate out-of-domain shifts.

**Fig. 10.**
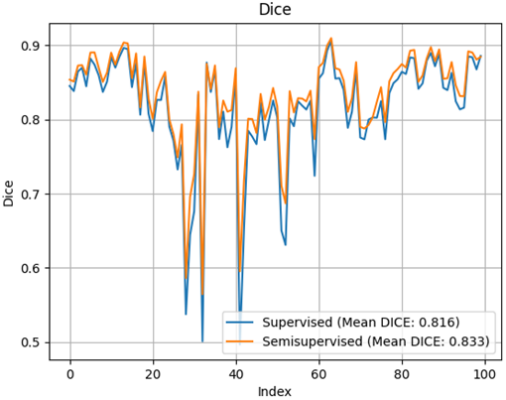
Dice value for the 100 images of the OASIS dataset. In orange the values for the semi-supervised approach showing higher mean and higher minimum values. Note that extreme cases show clear higher dice values.

Finally, results on OASIS dataset show a clear improvement on Dice index for the semi-supervised trained network (0.833 vs. 0.816). In Figure 10, the improvement for all 100 cases is shown visually, where we would like to highlight the enhancement in worst cases.

**Fig. 11.**
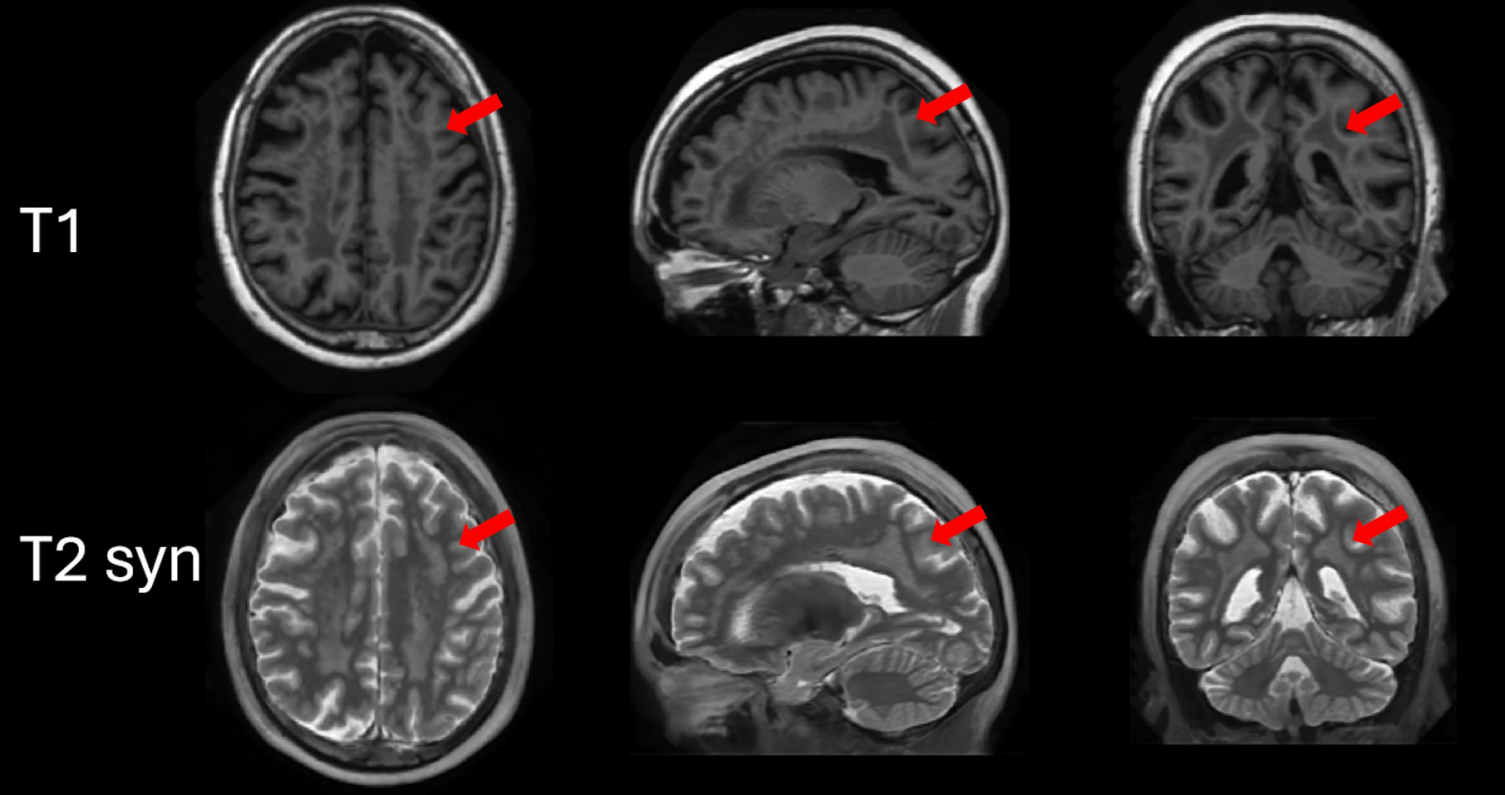
Qualitative results on the OASIS dataset. Top row: T1-weighted input images with visible white matter confluent lesions. Bottom row: synthesized T2-weighted images produced by the proposed method, showing consistent lesion depiction (red arrows).

Finally, to visually provide some evidence of the robustness of the proposed method, it was applied to OASIS images containing lesions. A visual example of the results of the proposed method is shown in Figure 11. As can be noticed, the lesions were correctly synthetized even though the supervised training data had no lesioned images.

### 3.4 State-of-the-art comparison

The proposed method was compared with related state-of-the-art related synthesis methods. To make a fair comparison, the code of the selected methods was downloaded and trained with our training library and tested with our test dataset. These include a 3D volume-based deep learning approach, a 2D diffusion-based generative model, and a slice-based generative adversarial network, having a representation of each technology. Below, we outline the details of each method:

- UNET3D (Manjón et al., 2022), this method employs a 3D volume-based deep learning approach using a stride-based variant of the U-Net architecture. The network processes entire 3D volumes, enabling it to capture spatial dependencies across slices effectively.
- SelfRDB (Arslan et al., 2024) is a diffusion-based generative model that leverages residual dense blocks (RDBs) for capturing hierarchical features during the synthesis process. Diffusion models excel at producing high-quality and realistic outputs by iteratively refining noisy initializations through a learned denoising process.
- The pGAN method (Dar et al., 2019) adopts a slice-based generative adversarial network architecture. Unlike 3D volume-based approaches, pGAN synthesizes slices independently, reducing the computational burden while maintaining high-quality outputs. This approach involves training a generator–discriminator pair, where the generator learns to produce realistic slices of data, and the discriminator refines the output by distinguishing real slices from generated ones. However, the slice-based nature of the approach can sometimes lead to discontinuities between slices in the final reconstructed volume.

When we compare the methods in our in-domain test data (n = 20 images), we can observe that for all metrics our method produces better results, followed by SelfRDB having the second-best Dice index (see Table 6).

**Table 6.** Results for in-domain test data (N = 20).

	PSNR	CC	SSIM	Dice
Our method	**32.2718** **±** 1.4842 *	**0.9869** **±** 0.0050 *	**0.9612** **±** 0.0097 *	**0.9095** **±** 0.0975 *
UNET3D	24.9035 ± 0.6391	0.9297 ± 0.0092	0.8251 ± 0.0159	0.7378 ± 0.2744
SelfRDB	28.4151 ± 4.6835	0.9458 ± 0.0479	0.8962 ± 0.0838	0.8166 ± 0.2435
pGAN	28.3846 ± 4.5307	0.9482 ± 0.0438	0.8810 ± 0.0884	0.6868 ± 0.2885

Best results in bold.

* Represents statistically significant differences between our method and the state-of-the-art methods.

Table 7 presents the results for the Bordeaux dataset, which was excluded from training for all methods, ensuring a more realistic assessment of performance. Notably, our model surpasses all competitors in every metric except SSIM, highlighting its superior image reconstruction capabilities and significantly improving its utility for downstream segmentation tasks.

**Table 7.** Results for out-domain Bordeaux test data (N = 44).

	PSNR	CC	SSIM	Dice
Our method	**28.3450** ± 0.7365* ^β^	**0.9715** **±** 0.0043* ^α^ ^β^	0.9152 ± 0.0116 *	**0.8583** **±** **0.0027** * ^α^ ^β^
UNET3D	25.5561 ± 0.6018	0.9426 ± 0.0049	0.8344 ± 0.0133	0.7481 ± 0.2794
SelfRDB	27.7835 ± 0.4756	0.9688 ± 0.0052	0.9160 ± 0.0101	0.8352 ± 0.1868
pGAN	27.7483 ± 0.9454	0.9688 ± 0.0056	**0.9165** **±** **0.0117** *^β^*	0.6700 ± 0.3091

Best results in bold.

* Represents statistically significant differences between our method and UNET3D

α Represents statistically significant differences between our method and SelfRDB.

β Represents statistically significant differences between our method and pGAN.

**Table 8.** Dice results in the OASIS test set (N = 100).

	Our method	UNET3D	SelfRDB	pGAN
Dice	**0.8350** **±** 0.0623*	0.6610 ± 0.0771	0.6641 ± 0.0929	0.6441 ± 0.1064

Best results in bold.

* Represents statistically significant differences between our method and the state-of-the-art methods.

**Fig. 12.**
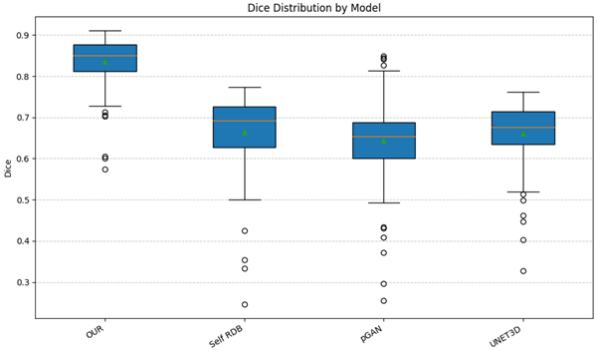
Dice distribution by model in OASIS dataset. Note that our proposed method has a lower dispersion than the other methods and the Dice index value is higher than the other state-of-the-art methods.

Finally, we also compared the different methods with the OASIS dataset (N = 100), which has the presence of lesions. Again, we indirectly assess the quality of the generated volume using the segmentation metrics as a proxy (see Table 8). We can also see the results in Figure 12, where we can see that the proposed method is more robust than the other methods (our method dispersion is lower than the other methods and the dice value is much higher).

**Fig. 13.**
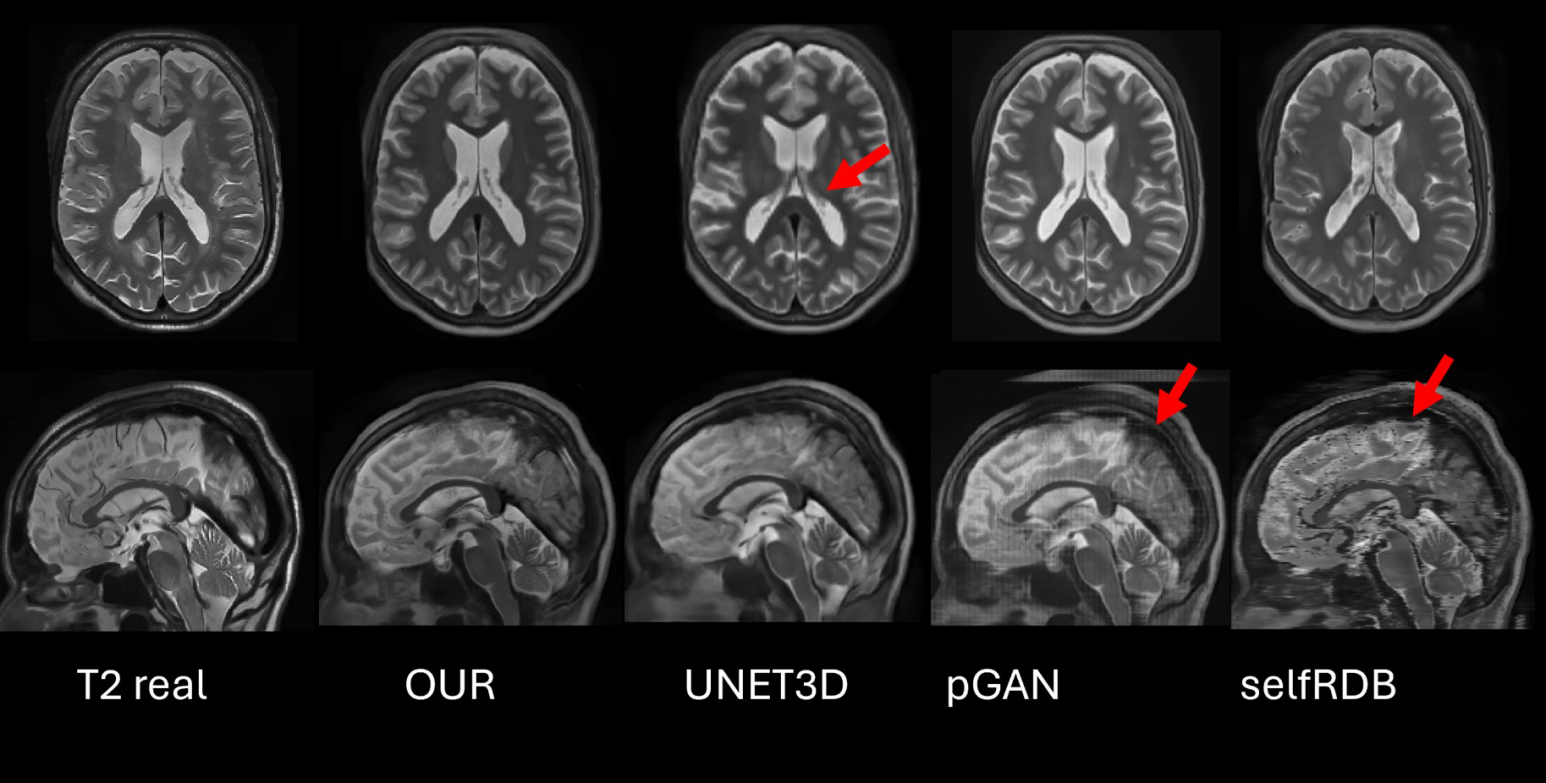
Visual comparison of methods using an example from the Bordeaux dataset. Red arrows in the top row indicate for UNET3D the inability to reconstruct the fine details from the choroid plexus within the lateral ventricles, in the bottom row, they indicate the artifacts in sagittal slice for pGAN and SelfRDB.

**Fig. 14.**
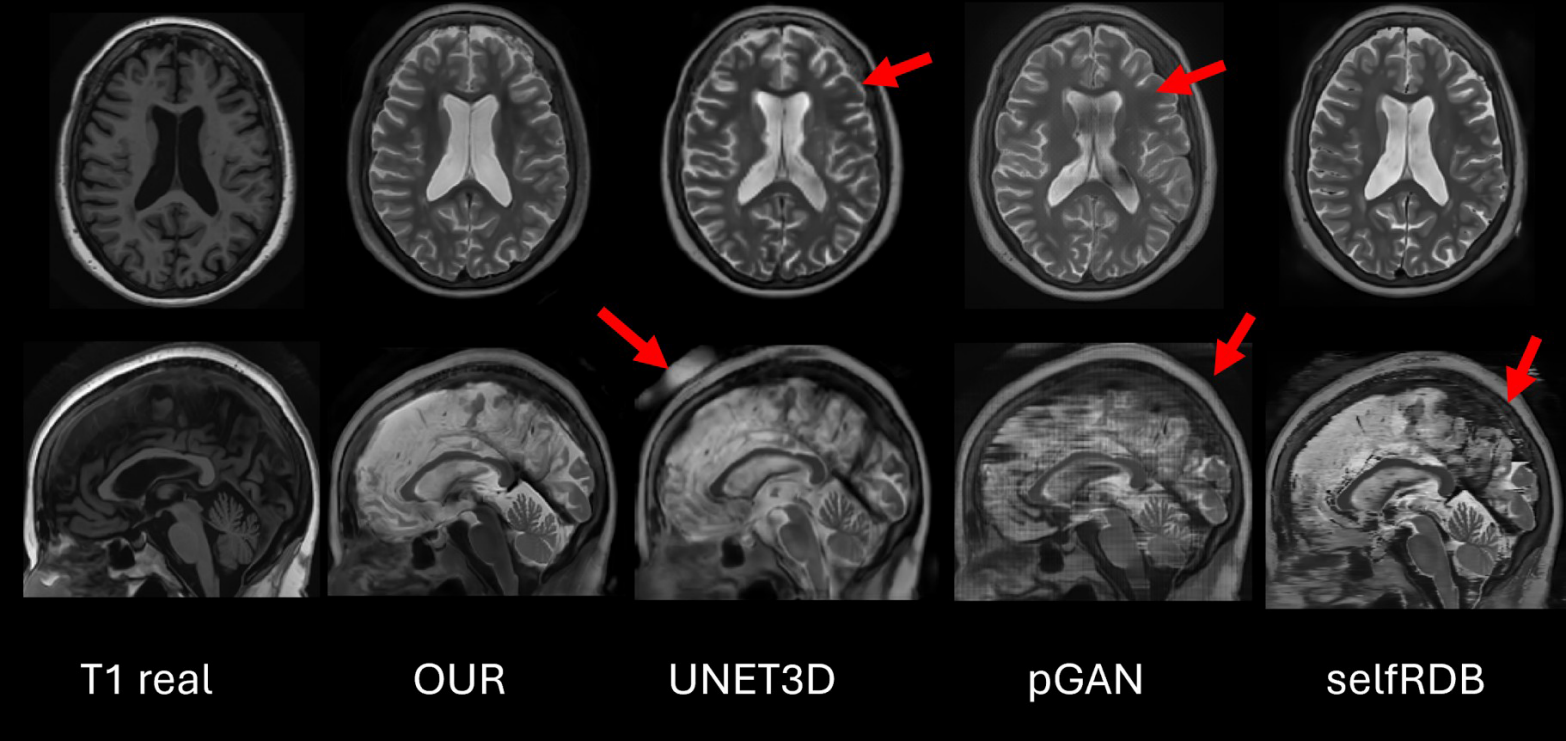
Comparison of the methods on an example of the OASIS dataset image. The real T1 is shown as a reference, as we do not have the T2 image for this dataset. Red arrows in the top row indicate fine details that cannot be well synthesized by the networks UNET3D and pGAN, in the bottom row, they indicate the artifacts in UNET3D and artifacts in pGAN and SelfRDB generated by obtaining the 3D image from the 2D slices.

To visually inspect the differences between the compared methods, in Figures 13 and 14 an example of the synthesized T2 volume for each method is shown. In the example of Bordeaux dataset (Fig. 13), the UNET3D method produces consistent but blurred images (probably due to the loss function used during its training). In addition, pGAN shows very realistic axial slices, but in the sagittal slice, pGAN has many artifacts, due to its 2D nature. Finally, SelfRDB produces convincing axial slices (with some vessel-like hallucinations) and (as pGAN) interslice artifacts due to its 2D nature. The proposed method shows a 3D coherent reconstruction and well-defined edges.

In the example case of OASIS dataset (Fig. 14), our proposed method and SelfRDB are the ones that generate better results, but again in the sagittal slice, SelfRDB has more artifacts and a worse image quality since it works in 2D and not in 3D as in our work.

A numerical analysis of these data indicates that the proposed method, along with pGAN and SelfRDB, performs competitively regarding classical metrics such as PSNR, SSIM, and CC. The key difference emerges when evaluating the Dice index, as discussed in Section 2.5. Here, the Dice index aims to assess visual quality in a more complex manner, aligning with the observations made from the reconstructed images. For instance, as shown in Table 7 (Bordeaux dataset) and Figure 13, pGAN achieves the highest SSIM performance. However, artifacts observed in the sagittal slice, which degrade visual quality of the reconstructed 3D volume, could explain the lower Dice index measured.

We finally compared the time required to train each model and their inference times. Regarding training time, in the case of the UNET3D (Manjón et al., 2022), the network was trained for 1,800 epochs, 50 steps per epoch and lasted 1 day. The training used the same dataset, preprocessing pipeline, and optimization settings as our method to ensure a fair comparison. SelfRDB (Arslan et al., 2024) was trained for 50 epochs (as suggested by the authors), requiring approximately 16 days to complete due to the computationally demanding nature of the 2D diffusion models. We trained pGAN for 100 epochs, each taking approximately 6,100 seconds, resulting in a total training time of around 7 days. The training time of our method lasts around 1 day, with 1,500 epochs. Regarding inference time, UNET3D time is around 1.5 seconds, and the Self RDB model takes around 3 minutes per case, making it significantly slower than most deep learning-based models. The inference time for pGAN was 16.5 seconds per case. Finally, the inference time of our proposed method is around 2 seconds. However, we must add the time to generate the atlas (T_atlas_ = 20 seconds), and the time to preprocess the image in terms of contrast stretching (T_tms_ = 3.8 seconds) resulting in an overall time of 25.8 seconds.

## 4 Discussion

In this paper, we have presented a novel deep learning-based method for synthesizing T2 MR images from T1 images. The proposed method works in 3D which eliminates interslice inconsistencies of previous 2D approaches and is designed with a segmentation-oriented loss function that improves the quality of the synthesized images.

We also explored incorporating prior anatomical information to enhance the model’s robustness and improve its performance on more complex images. This prior knowledge helps guide the synthesis process, allowing the model to handle anatomical variability better and ensure the generated images’ structural coherence. Our experiments demonstrate that models incorporating prior information outperform those relying solely on image-to-image translation, particularly in challenging cases with large lesions or advanced atrophy, such as cases with unusually large ventricles. Additionally, the proposed method incorporates a semi-supervised learning strategy, which enhances its robustness when applied to datasets with diverse characteristics, extending beyond the scope of the training data.

A critical component of the proposed method is the choice of loss function. To address this, we evaluated several loss functions, including those operating in both the spatial and frequency domains. We tested the mean squared error (MSE) of the Fast Fourier Transform (FFT) of the images, which captures global structural information that is often overlooked by spatial domain loss functions. Our results show that combining this frequency domain loss with a segmentation-oriented loss function best fits the problem. This hybrid approach allows the model to synthesize anatomical structures while ensuring predicted intensity consistency.

One of the key strengths of our method lies in its semi-supervised approach, which enables greater generalization across diverse datasets. The model demonstrates robustness because it has been trained on images spanning a wide range of age groups, from 3 years old and above, and a wide range of image types from 1.5T to 3T and with different gradient echo approaches such as MPRAGE of FSPGR. This adaptability is particularly relevant in clinical settings where datasets often vary significantly in terms of patient demographics and imaging protocols. One of the primary limitations in T2 synthesis tasks is the scarce availability of paired T1 and T2 images, which our semi-supervised approach is designed to address. While the use of prior information significantly mitigates challenges related to anatomical variability, we are aware that further efforts are needed to refine the method’s adaptability to extreme domain shifts or rare anatomical variations.

We compared the performance of our method against several state-of-the-art approaches, including SelfRDB (Arslan et al., 2024), a UNET3D-based method (Manjón et al., 2022) and a GAN-based method (Dar et al., 2019). The comparison highlights that our method is competitive, particularly in segmentation tasks. This improvement is attributed to the combined use of prior information, the segmentation loss function, and the frequency domain loss, which collectively enhances the synthesized image’s quality and utility for downstream tasks.

The proposed method has some practical implications. The improved Dice index and visual quality of the synthesized T2w MRI suggest that the proposed method can provide more accurate and clinically useful information, aiding in better diagnosis and treatment planning. The proposed method’s training and inference times are competitive, with training completed in approximately 1 day, inference taking around 2 seconds per case, and additional time for atlas generation and preprocessing.

Finally, we are aware that, as with every method, our method has its limitations. In order to simplify the synthesis process, we standardized the position and resolution of the images by mapping them to MNI space rather than using them at native space which can introduce some blurring due to the interpolation applied during registration. Besides, while the proposed method is efficient, the generation of the atlas and preprocessing steps add to the overall time, which could be optimized further. Although the model showed robustness across diverse datasets, further validation on more varied and larger datasets could strengthen the findings. Future work should include more extensive clinical validation and pathological modifications to assess the practical impact of the synthesized images on diagnostic accuracy and treatment outcomes.

## 5 Conclusion

We proposed a new synthesis method based on deep learning 3D CNN, specifically leveraging a combination of segmentation loss and prior information to guide the synthesis process. Unlike other studies that predominantly rely on generative adversarial networks (GANs) or diffusion models, we have developed a framework that avoids the instability typically associated with adversarial training and the large training/test times of diffusion approaches. Instead, our method focuses on integrating anatomical prior information, a powerful loss function and a semi-supervised learning paradigm, resulting in a more robust and generalizable solution.

Through experiments, we have validated the hypothesis that incorporating prior information and using loss functions tailored to the task, such as the Fourier transform loss combined with segmentation loss, significantly improves synthesis quality. The experiments demonstrated that our approach generates visually accurate T2 images and preserves anatomical and semantic consistency, even in datasets with diverse age ranges and anatomical variability.

Furthermore, the semi-supervised strategy proved critical in addressing the challenge of limited paired T1 and T2 datasets, enabling the model to generalize effectively to unseen data. This makes the proposed method especially useful in clinical scenarios where access to paired datasets is limited or infeasible.

Our results show that the proposed method outperforms state-of-the-art techniques, particularly in segmentation tasks, demonstrating its potential for applications that require accurate downstream analysis. In the future, we will explore contrast synthesis of other MR sequences such as FLAIR and Gd-enhanced T1, or modalities and their ability to detect small lesions.

## Data Availability

Public data are available online, while private data cannot be shared. Code will be available upon reasonable request.
